# Evaluation of the Ecological Environment Affected by *Cry1Ah1* in Poplar

**DOI:** 10.3390/life12111830

**Published:** 2022-11-09

**Authors:** Ali Movahedi, Hui Wei, Abdul Razak Alhassan, Raphael Dzinyela, Pu Wang, Weibo Sun, Qiang Zhuge, Chen Xu

**Affiliations:** 1 College of Biology and the Environment, Nanjing Forestry University, Nanjing 210037, China; 2 Key Laboratory of Landscape Plant Genetics and Breeding, School of Life Sciences, Nantong University, Nantong 226019, China; 3 Nanjing Key Laboratory of Quality and Safety of Agricultural Product, Nanjing Xiaozhuang University, Nanjing 211171, China

**Keywords:** *Bt* toxins, *Cry1Ah1* transgenic poplar, ecology, environment, rhizosphere

## Abstract

*Populus* is a genus of globally significant plantation trees used widely in industrial and agricultural production. Poplars are easily damaged by *Micromelalopha troglodyta* and *Hyphantria cunea*, resulting in decreasing quality. *Bt* toxin-encoded by the *Cry* gene has been widely adopted in poplar breeding because of its strong insect resistance. There is still no comprehensive and sufficient information about the effects of *Cry1Ah1*-modified (CM) poplars on the ecological environment. Here, we sampled the rhizosphere soils of field-grown CM and non-transgenic (NT) poplars and applied 16S rRNA and internal transcribed spacer amplicon Illumina MiSeq sequencing to determine the bacterial community associated with the CM and NT poplars. Based on the high-throughput sequencing of samples, we found that the predominant taxa included *Proteobacteria* (about 40% of the total bacteria), *Acidobacteria* (about 20% of the total bacteria), and *Actinobacteria* (about 20% of the total bacteria) collected from the natural rhizosphere of NT and CM poplars. In addition, studies on the microbial diversity of poplar showed that *Cry1Ah1* expression has no significant influence on rhizosphere soil alkaline nitrogen, but significantly affects soil phosphorus, soil microbial biomass nitrogen, and carbon. The results exhibited a similar bacterial community structure between CM varieties affected by the expression of *Cry1Ah1* and non-transgenic poplars. In addition, *Cry1Ah1* expression revealed no significant influence on the composition of rhizosphere microbiomes. These results broadly reflect the effect of the *Bt* toxin-encoded by *Cry1Ah1* on the ecology and environment and provide a clear path for researchers to continue research in this field in the future.

## 1. Introduction

Poplar (*Populus*) is a genus of globally important plantation trees used widely in industrial and agricultural production [1]. However, with the deterioration of the global environment, characterized by increasing salt, drought, pest, and disease stresses, the global production of poplar is becoming challenging. One approach to address this challenge is genetic modification. The manipulation of critical genes has been applied to alter poplar characteristics in transgenic lines, resulting in improved traits for better growth in adverse environments [2,3,4].

Insecticide resistance based on *Bacillus thuringiensis* (*Bt*) has allowed the development of a variety of insect resistance proteins for commercial genetically modified (GM) crops [5]. In addition, *Bt* toxin-encoded *Cry* genes have been widely applied in commercial GM crops, improving plant resistance to insect pests [6,7]. Despite the benefits of *Bt*-modified plants, a significant potential disadvantage is their effect on soil chemical properties and the structure and diversity of rhizosphere microorganisms, including bacteria and fungi [6]. Therefore, rhizosphere microorganisms associated with *Bt*-modified plants are highly interesting [8,9,10]. However, regarding the complexity of field conditions and the lack of a unified approach to microorganism analysis, studies exploring the influence of *Bt*-modified plants on soil microorganisms sometimes produce conflicting results [11,12,13,14]. For example, one study showed that soil microorganisms are not adversely affected by the cultivation of *Bt*-modified cotton plants [15].

In contrast, another study suggested that exogenous gene products expressed by Bt-modified transgenic crops may interact with soil microorganisms and affect their activities and functions [16]. *Bt*-modified plants have been found to alter bacterial population diversity compared with non-transgenic (NT) plants [17] and may enhance the population size of soil microbial communities [18,19]. Compared with NT rice, *Bt*-modified rice has been found to have a short-term effect on rhizosphere microbial community function [20]. Thus, there are some differences among studies on the impact of *Bt*-modified plant varieties on the soil microbial community. Further research is needed to evaluate the safety of *Bt*-modified crop plants. Since the commercialization of GM plants, the global planting area of GM crops has been overgrown, and new varieties of GM plants have been emerging continually [21]. GM plants have provided great economic and environmental benefits worldwide, such as increased plant yield and reduced chemical fertilizer and pesticide application. However, the potential impacts of GM plants on the ecological environment have raised concerns [22]. Soil microorganisms are essential to the soil ecosystem and involve various biochemical processes, including organic matter accumulation, mineralization, nutrient transformation, and circulation [23]. In addition, GM plants communicate with soil microorganisms during the growth process; therefore, research on the potential effects of GM plants on the soil microbial community is of great significance in evaluating their potential risks [24,25,26]. The study aimed to identify the effects of *Cry1Ah1*-modified (CM) poplar plantation on soil chemical properties and the diversity and structure of the soil microorganism community after three years of growth under field conditions. We applied high-throughput sequencing of 16S rRNA and internal transcribed spacer 1 (ITS1) to evaluate the diversity and composition differences in rhizosphere microorganisms between NT and CM varieties. The results contribute to our knowledge of how CM varieties affect rhizosphere soil microbial community function and provide reference information for evaluating the safety of CM varieties.

## 2. Materials and Methods

### 2.1. Plant Material and Experimental Field Design

In the previous study, the *Cry1Ah1* gene was cloned into the destination vector pH35GS. CM poplars, ’Nanlin 895’ (*Populus deltoides* × *Populus euramericana*), were regenerated by inoculating poplar leaf discs with Agrobacterium tumefaciens strain LBA4404, including recombinant plasmid pH35GS-*Cry1Ah1* [2]. To study the effects of CM poplars on a natural soil ecosystem, we designed a field test in Sihong, Jiangsu Province (118°68 N, 33°72 E). To identify the influence of CM varieties on the rhizosphere soil microbiome, we planted NT poplars in the experimental field. Three-year-old NT and CM varieties marked as A5-0, A4-6, Z1-3, A5-23, and A3-4 were selected, and six plots were established with four replicates per clone (Figure S1). An additional 3 m wide isolation zone was established between communities, with about 676 m^2^ (26 m × 26 m). The poplars were cultivated by cutting in March 2017 with permission from the State Forestry Administration. Moreover, the topography, physiognomy, soil, air temperature, vegetation, cultivation management, and other natural conditions were consistent. In addition, the experimental field was managed conventionally without chemical fertilizers or pesticides. The experimental field also confirmed similar soil characteristics and microenvironment.

There are four poplars in each small line area, including NT and CM varieties (lines A5-0, A4-6, Z1-3, A5-23, and A3-4) (Figure S1). The weeds and leaves were removed from the soil surface and a soil extractor was used to take out a soil column about 50 cm (diameter 8 cm) around every poplar rhizosphere. The fine roots in the 10–30 cm soil column were carefully taken out and the soil within 3 mm of fine roots was considered the rhizosphere soil. The rhizosphere soil of every four poplar trees in the small area was collected and mixed into one sample. The rhizosphere soil samples were taken out from the entire experimental field through this sampling method, and there were six duplicate rhizosphere soil samples for NT and each CM variety. All rhizosphere soil samples were passed through a 10-mesh sieve, mixed thoroughly, added to sterile centrifuge tubes, and then placed in a liquid nitrogen tank for transport to the laboratory.

### 2.2. Identification of Cry1Ah1 Expression Level and Insecticidal Activity

The fully expanded poplar leaves were collected from NT and CM varieties, and the collected samples were placed in a liquid nitrogen tank for transport to the laboratory. All the collected leaves, including NT and CM varieties, were assayed to detect *Cry1Ah1* expression levels using an ELISA kit (EnviroLogix, Portland, ME, USA). In addition, pupae of Micromelalopha troglodyta were collected from poplars cultivated in the field and hatched in a culture room at 27 ± 2 °C and 74% humidity with a 14 h light/10 h dark photoperiod. The eggs were collected from female adults and instar larvae were fed with NT and CM varieties. Larval mortality of M. troglodyta was counted on the 6th and 12th days. Three independent biological samples were performed.

### 2.3. Determination of Rhizosphere Soil Physical and Chemical Indices

Fumigation with chloroform was used to obtain the microbial biomass of nitrogen (MBN), microbial biomass of carbon (MBC), and microbial biomass of phosphorus (MBP) from the soil in the rhizosphere [12,27,28,29]. The rhizosphere soil samples treated with chloroform fumigation and non-chloroform fumigation were extracted with K2SO4 solution. The rhizosphere MBC, MBN, and MBP were measured using a Vario TOC cube analyzer (Elementar, Langenselbold, Germany). Briefly, fresh rhizosphere soil samples were dissolved in chloroform and the mixtures were boiled for 5 min in a vacuum. Then, 0.5 mol/L K2SO4 was added to the mixtures and subjected to fumigation in the dark at 25 °C for 24 h. The resulting mixture was then filtered with a quantitative filter paper. Simultaneously, the control groups were performed similarly, except that the rhizosphere soil samples were added to the reaction mixture for detection.

The rhizosphere soil alkaline nitrogen was determined using the Conway method (i.e., the alkali hydrolysis diffusion method). Briefly, 10 mL of NaOH was used to dissolve air-dried soil samples set in the outer chamber of a diffusion dish and 2 mL of boric acid (an indicator solution) was placed in the inner chamber. After incubation at 40 °C for 24 h, NH3 in the inner chamber absorption solution was titrated with 0.005 mol/L H2SO4 as a standard solution. Simultaneously, the control groups were performed similarly, except that the rhizosphere soil samples were added to the reaction mixture for detection.

The rhizosphere soil phosphorus was identified by molybdenum–antimony colorimetry. Briefly, air-dried soil samples were mixed with 0.5 mol/L NaHCO3 and activated carbon, shaken for 30 min, and filtered immediately with phosphate-free filter paper. Then, 1–5 mL of filtrate was extracted and the absorbance value was determined. Soil pH was also measured using the glass electrode method. Finally, the rhizosphere soil samples were mixed with 2.5× water volume and the suspension pH was determined using a PP-25 Professional Meter electrode (Sartorius, Germany). Simultaneously, the control groups were performed similarly, except that the rhizosphere soil samples were added to the reaction mixture for detection.

### 2.4. Rhizosphere Soil DNA Extraction and High-Throughput Sequencing

Using the Fast DNA Spin kit for soil (MP Biomedicals, Santa Ana, CA, USA), 36 independent rhizosphere soil samples were obtained from the NT poplars and five CM varieties (lines A5-0, A4-6, Z1-3, A5-23, and A3-4). Triplicate DNA extractions from each replicate of rhizosphere soil samples were mixed and composited into one DNA sample to overcome the heterogeneity. The quality and integrity of the DNA were determined by electrophoresis on 0.8% agarose gel and the extracted DNA samples were diluted 10-fold and stored at −80 °C for further molecular analyses. Using the extracted genomic DNA as a template, the V3-V4 region (515f/907r) of the 16S rRNA gene and ITS1 region (1737f/2043r) of the ITS1 rRNA gene were amplified to identify the composition and diversity of the microbiological community [30,31]. We then performed high-throughput sequencing using the Illumina novaseq platform. The raw data were filtered using the Trimmatic software to obtain high-quality clean paired-end reads, spliced using the FLASH software. The minimum overlap length was set to 10 bp and the maximum mismatch ratio of the splicing sequence was 0.1. After filtering, influential splicing segment clean tags were obtained. All clean tags were clustered using the VSEARCH software (https://github.com/torognes/vsearch, accessed on 12 April 2021). The clean tags were denoised in amplicon sequence variants (ASVs) and chimeras were filtered with UNOISE3. Taxonomic assignment of ASVs was performed in QIIME 2 v2018.2 [32] (https://qiime2.org, accessed on 12 April 2021) using the QIIME 2 feature classifier plugin [33].

All data analyses were performed using SPSS 19.0 software (IBM, Armonk, NY, USA). Differences in the physical and chemical properties between NT and CM varieties were evaluated using one-way analysis of variance (ANOVA) and Tukey’s post hoc comparison. The alpha diversity analyses were performed using the Chao1. It showed species indices to determine the community diversity of rhizosphere bacteria and the phylogenetic diversity (PD) whole-tree and Shannon indices to determine community richness and evenness [34]. According to ASVs’ clustering results, alpha diversity was calculated in Mothur v.1.30.1 with rarefaction analysis after subsampling the libraries to an exact size [35]. UniFrac was conducted with beta diversity analysis and phylogenetic analysis. Bray–Curtis distances between NT and CM varieties were calculated and visualized with principal component analysis (PCA).

## 3. Results

### 3.1. Effects of CM Varieties on M. Troglodyta

The Bt-Cry1A ELISA kit was used to identify the *Cry1Ah1* expression level in NT and CM varieties. The results showed that *Cry1Ah1* was expressed in CM varieties, and lines A4-6 and A5-0 had a higher *Cry1Ah1* expression level. Conversely, line A3-4 had a lower *Cry1Ah1* expression level (Table S1). In addition, the insecticidal activities of CM varieties were identified, and the results showed that the CM varieties had higher insecticidal activity to *M. troglodyta* than NT poplars (Figure S2). Significantly, lines A4-6 and A5-0 with higher *Cry1Ah1* expression levels exhibited relatively more substantial insecticidal activity than *M. troglodyta*.

### 3.2. Effects of CM Poplars on Rhizosphere Soil Chemistry Patterns

**Figure 1:**
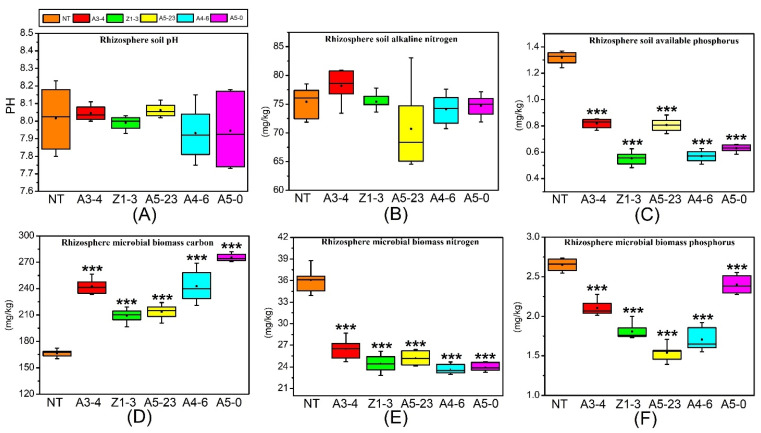
Evaluation of rhizosphere soil physical and chemical properties in non-transgenic (NT) and *Cry1Ah1*-modified (CM) poplar varieties. Analysis of rhizosphere soil pH (**A**), rhizosphere soil alkaline nitrogen (**B**), rhizosphere soil phosphorus (**C**), rhizosphere microbial biomass carbon (**D**), rhizosphere microbial biomass nitrogen (**E**), and rhizosphere microbial biomass phosphorus (**F**) in NT and CM poplar varieties. Data were analyzed using one-way analysis of variance (ANOVA) and Tukey’s post hoc comparison. *** *p* < 0.001.

During the first three years of poplar establishment, the mean soil pH ranged from 7.73 to 8.23 in rhizosphere soil (Figure 1A). Moreover, there was no significant change in rhizosphere soil pH between NT and CM varieties by the sampling date. For the CM varieties (lines A5-0, A4-6, Z1-3, A5-23, and A3-4), rhizosphere soil alkaline nitrogen ranged from 64.54 to 83.15 mg/kg, with similar values observed for NT poplars, and no significant difference between NT and CM varieties (Figure 1B). However, the CM varieties had significantly lower rhizosphere soil available phosphorus in the field-grown stage than NT poplars (Figure 1C). The rhizosphere MBC contents of NT poplars ranged from 160 to 172 mg/kg and differed significantly from those of the CM varieties (Figure 1D). In addition, CM varieties had significantly lower MBN and MBP contents than NT poplars (Figure 1E,F).

### 3.3. Data Quality Control and ASVs’ Analysis

Using the Illumina hiseq, an average of 53,567 and 68,783 16S rDNA tags and 33,750 and 87,922 ITS1 tags were generated from the rhizosphere microbiome. Chimeras and short tag sequences were removed to obtain high-quality clean tags comprising an average of 33,390 and 86,787 16S rDNA tags and 21,523 and 22,555 ITS1 tags (Table S2). Moreover, clean tag distributions of rhizosphere bacteria were visualized. The results showed that clean tags ranged from 200 to 440 bp and clean tags with lengths of 420 to 440 bp occupied the largest proportion (Figure S3A). In contrast, clean tag distributions of rhizosphere fungi ranged from 200 to 360 bp and clean tags with lengths of 200 to 260 bp occupied the largest proportion (Figure S3B). Using Qiime ver. 2.0 and Vsearch 2.7.1, the chimeric and organelle sequences were removed to produce 10,787 rhizosphere bacterial community sequencing ASVs and 7732 fungal community sequencing ASVs (Table S3).

### 3.4. Rhizosphere Bacterial Diversity

**Figure 2:**
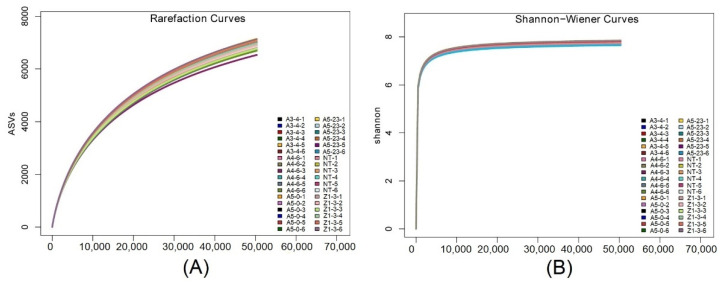
Based on the results of ASV clustering, the rarefaction of ASV has been analyzed. (**A**), Differences in the diversity of the rhizosphere bacterial community between NT and CM varieties have been shown. (**B**), The diversity of the rhizosphere bacteria from NT and CM has been compared by Shannon-Wiener curves.

To construct alpha rarefaction curves and evaluate the putative differences in the alpha diversity, the mothur was applied to perform ASV rarefaction analysis based on ASV clustering results. The sample rarefaction curves of rhizosphere bacteria illustrated that most NT and CM varieties saturate around 6500–7000 ASVs, suggesting slight differences in the diversity of the rhizosphere bacterial community between NT and CM varieties (Figure 2A). The Shannon–Wiener curves were also constructed to evaluate the rhizosphere bacterial diversity. The results showed that the Shannon curves are flat when the number of reads reaches 10,000, illustrating that the amount of sequencing data is sufficient to reflect the vast majority of rhizosphere bacterial information in the 36 samples. In addition, Shannon curves of the 36 samples fitted together (Figure 2B) suggested that rhizosphere bacterial communities in different sequencing depths share similar diversity.

**Figure 3:**
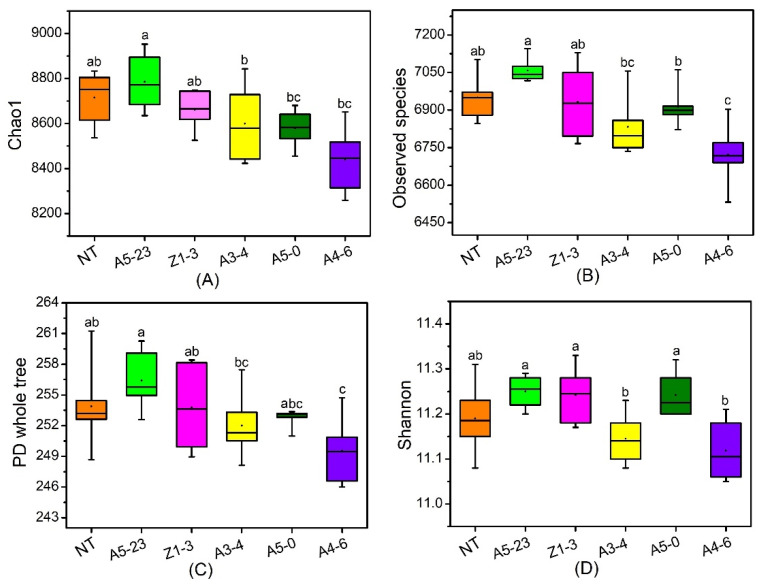
The analysis of the taxonomic distinctiveness of rhizosphere bacteria has been presented based on the alpha diversity as (**A**) the Chao1 index, (**B**) the observed species index, (**C**) the phylogenetic diversity (PD) whole-tree index, and (D) the Shannon index. Data were analyzed using one-way ANOVA and Tukey’s post hoc comparison. Significant differences (*p* < 0.05) are indicated in lowercase letters. The NT, A5-23, Z1-3, A3-4, A5-0, and A4-6 lines were represented by orange, green, fusica, yellow, juniper, and violet rectangles, respectively.

The alpha diversity analysis was used to reflect the richness and diversity of rhizosphere bacteria. The Chao1 indexes in NT poplars had highly similar results in CM varieties, including A5-23, Z1-3, and A3-4. At the same time, the *Cry1Ah1* expression might increase the community richness of rhizosphere bacteria, for which the analysis of Chao1 showed no dominant difference between NT and CM varieties (Figure 3A and Table S4). Moreover, the observed species in NT poplars had no dominant differences compared with CM varieties, except for line A4-6 (Figure 3B and Table S4). The PD whole tree in NT and CM varieties shared similar features with the observed species (Figure 3C and Table S4). There are no significant differences in the analysis of the Shannon curves (Figure 3C,D and Table S4), which suggests that the community diversity of rhizosphere bacteria was similar between NT and CM varieties. Based on the alpha diversity, we concluded that *Cry1Ah1* expression slightly influences the rhizosphere bacterial richness, but does not affect the community diversity of rhizosphere bacteria.

**Figure 4:**
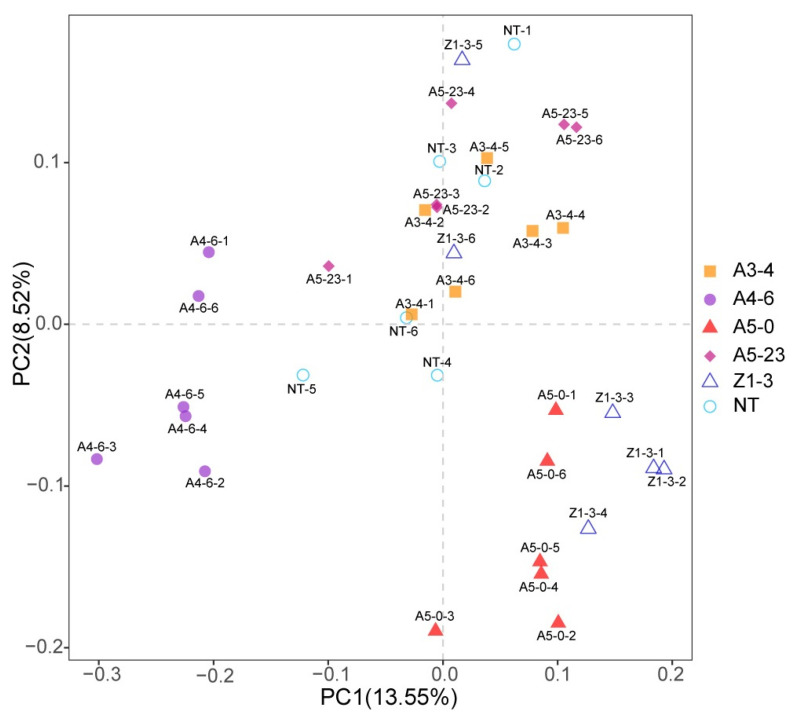
Principal component analysis (PCA) of rhizosphere bacterial communities at the ASVs level. ASVs were defined at a 97% sequence similarity cut-off in mothur. The differences and distances among NT, A5-0, A4-6, Z1-3, A5-23, and A3-4 can be visualized based on an analysis of ASVs’ composition.

A Bray–Curtis dissimilarity matrix was calculated on normalized and square-root-transformed read abundance data to compare the composition of rhizosphere bacterial members between NT and CM varieties. Based on weighted UniFrac, beta diversity analysis with PCA was applied to analyze the bacterial community structures among NT, A5-0, A4-6, Z1-3, and A5-23, and A5-0, A4-6, Z1-3, A5-23, A3-4, and NT overlapped each other and could not be separated (Figure 4), which indicated that the community structures of NT and CM varieties were similar. The *Cry1Ah1* expression did not affect the bacterial community structures.

### 3.5. The Higher Cry1Ah1 Expression Level May Have Marginal Effects on Rhizosphere Bacteria of Field-Grown Poplars

**Figure 5:**
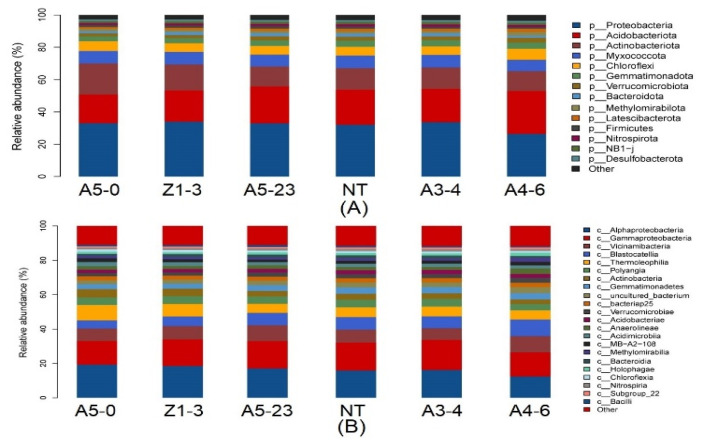
The overall composition of rhizosphere bacterial communities and the relative abundances of rhizosphere bacteria at the phylum or class level between NT and CM varieties. Phylum-level (**A**) and class-level (**B**) taxonomic analysis of bacterial distribution in rhizosphere soil samples of the NT and CM varieties based on 16S amplicon data.

**Figure 6:**
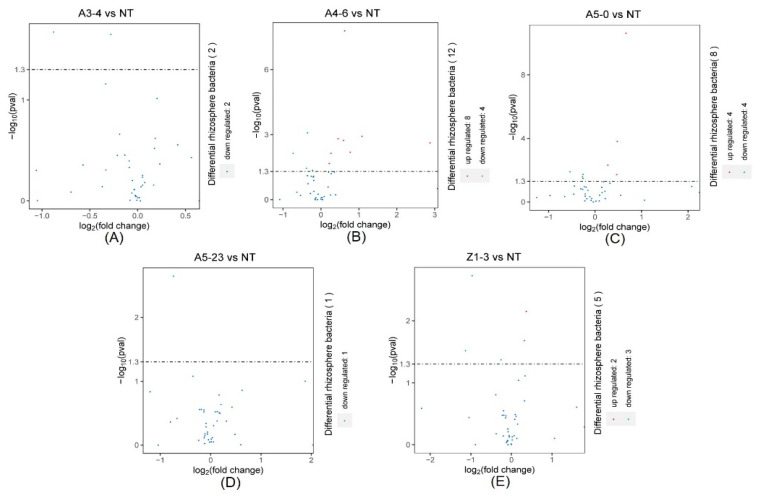
The DeSeq2 analysis for the selection of statistically differential rhizosphere bacteria at the phylum level between NT and lines A3-4 (**A**), A4-6 (**B**), A5-0 (**C**), A5-23 (**D**), and Z1-3 (**E**).

We investigated the taxonomic distinctiveness of poplar rhizosphere bacteria to determine the effect of *Cry1Ah1* expression on the rhizosphere bacteria. In addition, the DeSeq2 was used to select the putative statistically differential rhizosphere bacteria. The relative abundances of NT and CM varieties of rhizosphere bacteria at the phylum, class, order, family, and genus levels were identified. At the phylum level, Firmicutes, Myxococcota, Nitrospirota, Sva0485, Fibrobacterota, Latescibacterota, Desulfobacterota, and Proteobacteria, considered the dominant bacteria, were found in the rhizosphere bacterial community (Figure 5A). The DeSeq2 analysis found that the dominant bacteria share similar abundances between NT and CM varieties. In contrast, the relative abundances of Cyanobacteria and Methylomirabilota showed a significant difference between NT and line A3-4 (Figure 6A). Moreover, Methylomirabilota, Proteobacteria, Zixibacteria, MBNT15, Dadabacteria, Thermoplasmatota, Cyanobacteria, Chloroflexi, Bacteroidota, Acidobacteriota, Firmicutes, and Myxococcota were present at different abundances between NT and line A4-6 (Figure 6B). In addition, a few rhizosphere bacteria abundances were the difference between NT and A5-0, A5-23, or Z1-3 (Figure 6C–E). According to the above evidence, *Cry1Ah1* expression does not influence most rhizosphere bacteria abundances and only changes a small part of rhizosphere bacteria abundances.

**Figure 7:**
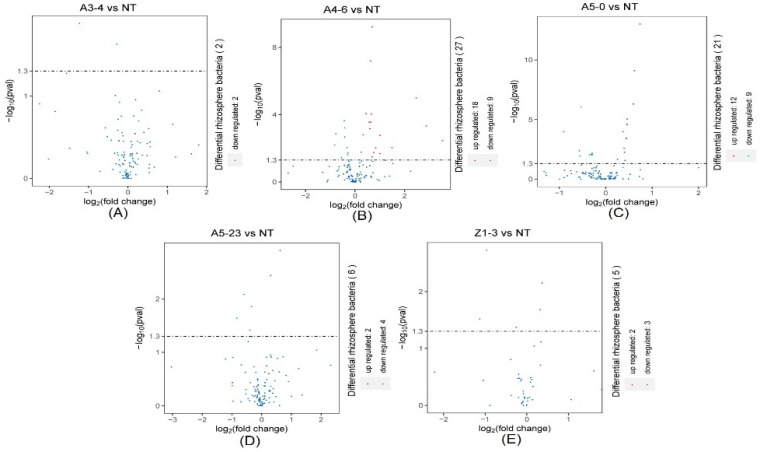
The DeSeq2 analysis for the selection of statistically differential rhizosphere bacteria at a class level between NT and lines A3-4 (**A**), A4-6 (**B**), A5-0 (**C**), A5-23 (**D**), and Z1-3 (**E**).

At the class level, the rhizosphere bacterial community composition of NT and CM varieties was similar (Figure 5B). The relative abundances of rhizosphere bacteria had no significant difference between NT and line A3-4, except for Cyanobacteria and Methylomirabilia (Figure 7A).

**Figure 8:**
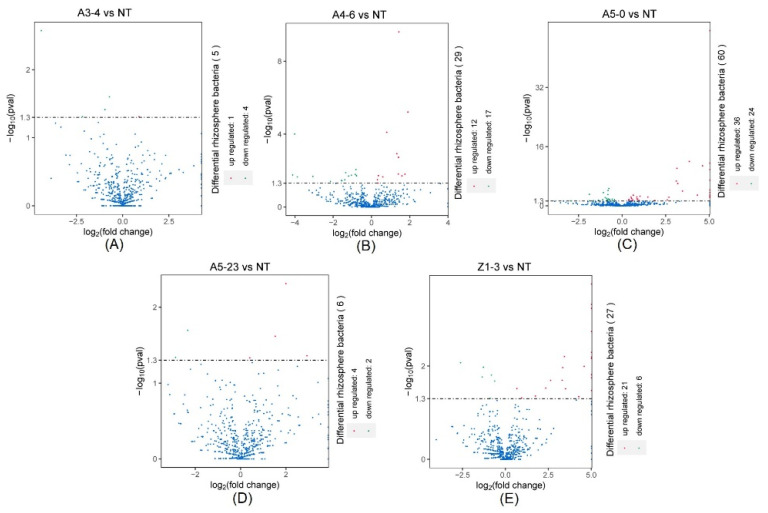
The DeSeq2 analysis for the selection of statistically differential rhizosphere bacteria at the species level between NT and lines A3-4 (**A**), A4-6 (**B**), A5-0 (**C**), A5-23 (**D**), and Z1-3 (**E**).

In native fields, Alphaproteobacteria, Deltaproteobacteria, Betaproteobacteria, Subgroup6, Blastocatellia, and Thermoleophilia accounted for approximately 60% of the total rhizosphere bacteria and were present at similar relative abundances in NT and CM varieties (Figure 5B). In addition, rhizosphere bacteria with lower relative abundances, such as Actinobacteria, Gemmatimonadetes, Nitrospira, Holophagae, and Acidimicrobiia, were significantly different between NT and line A4-6 (Figure 7B). Compared with NT, the relative abundances of rhizosphere bacteria in lines A5-0, A5-23, and Z1-3 were similar and only small parts of rhizosphere bacteria abundances displayed differences (Figure 7C–E). At the species level, a minor part of rhizosphere bacteria abundances from NT was slightly lower or higher than the CM varieties, indicating that the CM varieties had little influence on rhizosphere bacteria with lower relative abundances (Figure 8). We investigated the taxonomic distinctiveness of poplar rhizosphere soil fungi to determine whether *Cry1Ah1* expression affected rhizosphere fungus communities. The Chao1 analysis showed that the community richness of rhizosphere fungi in CM varieties shares a similar community richness to NT, except for line A5-0 (Figure S4A). In addition, no significant difference was present in the observed species, PD whole tree, and Shannon between NT and CM varieties. However, the observed species and PD whole tree in line A5-23 had no slight difference in line Z1-3 (Figure S4B–D). The alpha diversity showed that *Cry1Ah1* expression may slightly improve the fungal community richness, but does not influence the diversity of rhizosphere fungi. PCA was used to evaluate the fungal community structures among NT and CM varieties based on the Bray–Curtis dissimilarity matrix. The results showed that NT and CM varieties are gathered together and the *Cry1Ah1* expression does not affect the fungal community structures (Figure S5). The dominant fungal phyla in poplar rhizosphere soils included Ascomycota, Basidiomycota, and Mortierellomycota (Figure S6A); the sequence load of the six dominant phyla, represented by high sequence numbers, represented more than approximately 80% of the total sequence, whereas that of low-abundance phyla comprised less than 20% of the entire sequence (Figure S6A). Except for Ascomycota, there was no significant difference among rhizosphere fungi between NT and CM varieties (Table 1). Based on the analysis of rhizosphere fungal abundance in NT and CM varieties, we concluded that *Cry1Ah1* expression has no significant influence on the relative abundances of most rhizosphere fungi and only affects a few rhizosphere fungal abundances. We filtered extremely rare ASVs from the dataset to determine relative abundances at the class, order, family, and genus levels.

Similar relative abundances of most rhizosphere fungi were observed between NT and CM varieties (Figure S6B–E). However, the relative abundances of Archaeosporomycetes, Agaricostilbomycetes, Lobulomycetes, Cystobasidiomycetes, Schizosaccharomycetes, Sordariomycetes, and Ascomycota Incertae sedis at the class level were different between NT and CM varieties (Table S5). Compared with NT poplars, only 13 kinds of 148 rhizosphere fungi relative abundances in CM varieties were different (Table S6). Moreover, the major rhizosphere fungi at the family level showed similar abundances between NT and CM varieties. In contrast, only 7.9% of rhizosphere fungi with a lower abundance were present at differences between NT and CM varieties (Table S7). In addition, most rhizosphere fungi abundances were found to have no differences between NT and CM varieties at the genus level (Table S8). The rhizosphere fungal abundance analysis indicated that most rhizosphere fungi are low and not significantly different between NT and CM varieties. Only relative abundances of a few rhizosphere fungi were different in NT poplars and CM varieties.

## 4. Discussion

Biological diversity comprises community composition, structure, and function [36]. Interactions between soil microorganisms and other organisms influence nutrient cycling, which is essential in soil condition, quality, and health [12,37,38]. The rhizosphere is a functional interface for material exchange between plants and soil ecosystems. Plants assimilate CO_2_ during photosynthesis and transport some photosynthetic products to their underground parts, promoting the growth and metabolism of soil microorganisms, which transform organic nutrients into inorganic forms for plant absorption and utilization [39]. With the recent emergence of transgenic plants, the impact of their cultivation on the structure and function of the rhizosphere microbial community has become a concern [16,40,41]. Therefore, the structural diversity of the rhizosphere microbial community is an essential index for evaluating the effects of GM on the soil ecological environment.

### 4.1. Changes in Rhizosphere Soil MBC, MBN, and MBP Content in NT and CM Varieties

The rhizosphere soil microbial biomass is essential for assessing active soil nutrients and a sensitive indicator of environmental change in terrestrial ecosystems [42,43,44]. In addition, MBC, MBN, and MBP participate in the ecosystem cycling of carbon, nitrogen, and phosphorus [45,46]. However, the effects of GM plants on MBC, MBN, and MBP have not been reported. Therefore, in the present study, we examined the impact of field-cultivated CM plants on MBC, MBN, and MBP contents to study the relationship between CM plant growth and carbon, nitrogen, and phosphorus transformation in natural soil. As a part of active soil carbon, MBC is the driving force of soil organic matter decomposition, closely related to the cycling of soil elements. We found that rhizosphere MBC content is significantly higher in CM varieties than in NT poplars.

Conversely, rhizosphere MBN content decreased in CM varieties grown in the field, affecting rhizosphere microorganisms’ growth, metabolism, and structure. As an essential source of active soil nitrogen, MBN plays a vital role in regulating soil nitrogen supply [47]. In addition, MBP is the most active component of soil organic phosphorus, which governs the mineralization and fixation of soil phosphorus. Thus, MBP is an essential source of soil phosphorus, reflecting functional capacity and turnover intensity [48,49]. Our results showed that CM varieties alter the rhizosphere MBN and MBP contents, at least during the study period, affecting the capacity of soil microorganisms to metabolize carbon, nitrogen, and phosphorus.

The rhizosphere MBC/MBN ratio can reflect the rhizosphere microbial community structure. The MBC/MBN ratio is about 5:1 for bacteria, 6:1 for actinomycetes, and 10:1 for fungi [50,51,52,53,54]. Based on our results, the MBC/MBN ratio for NT poplars in our study site was about 4.6 ± 0.3, indicating that rhizosphere bacteria may play a dominant role in determining rhizosphere MBC and MBN contents. However, the MBC/MBN ratio for CM varieties was about 9.2 ± 1.7, suggesting that rhizosphere microbial fungi in CM varieties participate widely in rhizosphere soil microenvironment regulation. Xu et al. [30] systematically analyzed MBC, MBN, and MBP in the global terrestrial ecosystem. They reported mean values of MBC/MBN, MBC/MBP, and MBN/MBP ratios of 7.6, 42.4, and 5.6. In the present study, the rhizosphere MBC/MBP and rhizosphere MBN/MBP ratios for NT poplars were 63.2 ± 3.1 and 13.6 ± 0.9, respectively, higher than those reported for the global terrestrial ecosystem. This discrepancy may be because of the low nitrogen and phosphorus content in the experimental field, resulting in lower rhizosphere MBN and rhizosphere MBP contents and lower rhizosphere MBC/MBP and rhizosphere MBN/MBP ratios. Compared with NT poplars, CM varieties showed higher rhizosphere MBC/MBN, rhizosphere MBC/MBP, and rhizosphere MBN/MBP ratios; thus, *Cry1Ah1* transformation may directly affect the growth of rhizosphere microorganisms or inhibit rhizosphere microbial activity, thereby affecting the metabolism of MBC, MBN, and MBP.

### 4.2. Effects of Cry1Ah1 Expression on Native Rhizosphere Communities

Bt protein confers strong insecticide resistance as a dominant trait of GM crops; it has been widely used in transgenic breeding to achieve insecticide-resistant plants. Xu et al. [2] showed that field-planted CM poplars had strong insecticide resistance. With increasing GM crops worldwide, GM crop cultivations’ environmental and ecological impact has raised concerns globally. Some studies have shown that GM crops seriously affect biodiversity and threaten the environment [55]. Whether GM crops affect rhizosphere microbial composition, structure, and function has become a widely studied question in oncology and food safety [56]. The plant rhizosphere is a dynamic microenvironment in which many factors, such as plant species, soil type, and root location [57,58,59], affect the composition and structure of microbial communities around plant roots [60]. Therefore, to avoid the interference of these factors in examining rhizosphere microbial communities in the present study, we selected poplar trees planted in a single location and collected samples simultaneously. Such a design can effectively avoid the influence of other factors on the results, focusing only on the effects of poplar type (NT or CM) on the rhizosphere microbial community.

Many studies have explored the relationship between soil biodiversity and the ecological safety of transgenic plants. The high insect resistance of Bt-maize makes it an important transgenic crop and it has been found not to affect soil microbial communities [61] or rhizosphere communities [6]. Field-cultivated *Bt* transgenic cotton has no significant effect on rhizosphere communities other than WT cotton [62]. In the present study, the community diversity and rhizosphere bacterial abundances in NT and CM varieties showed no significant difference. Similarly, Cry1Ac-sugarcane was found to have no impact on rhizosphere microbial diversity or enzyme activity compared with NT sugarcane within a single crop season [63]. Furthermore, NT and Bt-modified rice detected no persistent or adverse effects on the rhizosphere bacterial community population [64]. In addition, Li et al. [15] reported that Bt transgenic rice could change bacterial community composition but not fungal abundance or community structure. Some communities contained a few dominant taxa, whereas others contained many low-abundance taxa. According to the alpha diversity, we found the observed species and PD whole tree differ between NT and line A4-6, which suggested a difference in species richness and community diversity between NT and line A4-6. Concerning the subsequent analysis of relative abundances of rhizosphere bacteria in NT and CM varieties, we concluded that the difference in species richness and community diversity might have originated from the rhizosphere bacteria with low abundance. Weinhold et al. [65] performed a similarity percentage analysis to identify major differences in abundance within groups and found that highly abundant families contributed significantly to dissimilarities. Therefore, according to the alpha diversity, *Cry1Ah1* expression had no significant influence on the rhizosphere bacterial richness and community diversity of rhizosphere bacteria. Based on identifying relative abundances of rhizosphere bacteria in NT and CM varieties, we found no significant difference in the abundances of major rhizosphere bacteria between NT and CM varieties, while the only differences were found in the minor rhizosphere bacteria between NT and CM varieties. We also concluded that the *Cry1Ah1* expression does not affect the relative abundances of major rhizosphere bacteria in native fields and *Cry1Ah1* expression had no impact on most rhizosphere fungal abundances. Moreover, the taxonomic diversity and structure of rhizosphere fungal communities and the relative abundances of most rhizosphere fungi were similar among NT and CM varieties. However, a small fraction of rhizosphere fungal abundances in special CM varieties differed from those in NT poplars. Based on these findings, we concluded that *Cry1Ah1* expression has no effect on the rhizosphere microbial community composition and large numbers of microbial abundances.

## 5. Conclusions

This study revealed no significant effects of NT and CM cultivation on microbial population and community structure; meanwhile, most rhizosphere bacteria shared similar relative abundances between the NT and CM varieties, suggesting that *Cry1Ah1* expression has no effects on the major rhizosphere bacteria abundances. In conclusion, *Cry1Ah1* expression has no change in microbial population and community structure and does not impact most rhizosphere bacterial abundances.

## Figures and Tables

**Table 1:** The relative abundances of rhizosphere fungi at the phylum level between NT and CM varieties. Data were analyzed using the Kruskal–Wallis comparison. “*p* < 0.05” indicates a significant difference between NT and CM varieties.

ASV	Test-Statistic	*p*	FDR P	Bonferroni P	A5-0 Mean	NT Mean	A5-23 Mean	A4-6 Mean	A3-4 Mean	Z1-3 Mean
p__Ascomycota	13.56156156	0.018647881	0.354309737	0.354309737	0.38735852	0.61138326	0.61042142	0.243904621	0.484990829	0.392447323
p__Basidiomycota	10.16216216	0.070768701	0.391712711	1	0.227967163	0.135322999	0.253847358	0.417125218	0.331415246	0.198798819
p__Chlorophyta	10.15615616	0.070929628	0.391712711	1	0.01805686	0.011899969	0.006559522	0.009774974	0.01853778	0.021434483
p__Chytridiomycota	9.092451776	0.105432904	0.391712711	1	0.001884535	0.003696372	0.001263812	0.000542433	0.000810853	0.002152955
p__Cercozoa	8.697065259	0.121774576	0.391712711	1	0.045888695	0.017676598	0.006710509	0.007348007	0.007040442	0.00904241
p__unidentified	8.156156156	0.147836947	0.391712711	1	0.159939382	0.07572809	0.076952758	0.25286315	0.111400036	0.232972084
p__Entomophthoromycota	7.806407963	0.167232549	0.391712711	1	0.000117434	0.000849998	0.000995392	6.15 × 10^−5^	6.15 × 10^−5^	3.36 × 10^−5^
p__Rozellomycota	7.579196217	0.181002706	0.391712711	1	0.000380262	0.001314141	0.000665459	0.000687827	0.00061513	0.001101642
p__Mortierellomycota	7.507507508	0.185548126	0.391712711	1	0.13757102	0.115996734	0.026478549	0.028184136	0.020472643	0.116136536
p__Blastocladiomycota	6.846918489	0.232276395	0.44132515	1	0.003265781	0.001213484	4.47 × 10^−5^	0.003282557	0.00246052	0.001302957
p__Olpidiomycota	5.728450555	0.333544833	0.537634093	1	0.000866774	5.03 × 10^−5^	7.83 × 10^−5^	0.000111842	0.000117434	0.000123026
p__Zoopagomycota	5.322944896	0.377751263	0.537634093	1	0.000715788	0.002024337	0.000419407	0.000726972	0.000726972	0.001588154
p__Glomeromycota	5.162162162	0.396412153	0.537634093	1	0.01409766	0.021809153	0.014824632	0.034631817	0.017508836	0.021311457
p__Entorrhizomycota	5	0.415880187	0.537634093	1	0	0	1.12 × 10^−5^	0	0	0
p__Monoblepharomycota	4.930281072	0.424447968	0.537634093	1	0.000352302	0.000335525	6.71 × 10^−5^	4.47 × 10^−5^	0.000173355	0.000313157
p__Ciliophora	3.628726784	0.60400532	0.61504672	1	0.000363486	1.12 × 10^−5^	4.47 × 10^−5^	5.59 × 10^−6^	0.002829598	0.000184539
p__GS19	3.627922155	0.604125841	0.61504672	1	2.80 × 10^−5^	0	2.24 × 10^−5^	0	3.91 × 10^−5^	5.59 × 10^−6^
p__Kickxellomycota	3.615658975	0.605963711	0.61504672	1	0.00096184	0.000548025	0.00048092	0.000570393	0.000609538	0.00072138
p__Mucoromycota	3.555235853	0.61504672	0.61504672	1	0.000184539	0.000139802	0.000111842	0.00013421	0.000190131	0.000329933

## Data Availability

Not applicable.

## Supplementary Materials

The following supporting information can be downloaded at https://www.mdpi.com/article/10.3390/life12111830/s1. Figure S1: Poplar growth and experimental design for poplar field trials. Diagram of the experimental design for poplar field trials. The NT and CM varieties were arranged randomly. The CM varieties are A5-0, A4-6, Z1-3, A5-23, and A3-4. Figure S2: Insecticidal activity of NT and CM varieties against Micromelalopha troglodyta. The larval mortality of *M. troglodyta* on days 6 (A) and 12 (B) of feeding on NT and CM varieties. Data were analyzed using one-way ANOVA and Tukey’s post hoc comparison. * *p* < 0.05 and ** *p* < 0.01, respectively. Figure S3: The distribution of rhizosphere microbe clean tags. The clean tags’ distribution of rhizosphere bacteria (A) and rhizosphere fungi (B). Figure S4: Analysis of the taxonomic distinctiveness of rhizosphere fungi based on alpha diversity. (A) Chao1 index. (B) Observed species index. (C) Phylogenetic diversity whole-tree index. (D) Shannon index. Data were analyzed using one-way ANOVA and Tukey’s post hoc comparison. Significant differences (*p* < 0.05) are indicated with lowercase letters. Figure S5: PCA of rhizosphere fungal communities at the level of ASVs. ASVs were defined at a 97% sequence similarity cut-off in mothur. The differences and distances among NT, A5-0, A4-6, Z1-3, A5-23, and A3-4 can be visualized based on an analysis of ASVs’ composition. Figure S6: Overall composition of rhizosphere fungal communities and the relative abundances of rhizosphere bacteria between NT and CM varieties. Phylum-level (A), class-level (B), order-level (C), family-level (D), and genus-level (E) taxonomic analysis of fungal distribution in rhizosphere soil samples of the NT and CM varieties based on ITS1 amplicon data. Table S1: Analysis of *Cry1Ah1* expression level in NT and CM varieties. Table S2: The 16S rDNA tags and ITS1 tags generated from the rhizosphere microbiome. Table S3: ASVs for bacterial and fungal community sequencing. Table S4: The alpha diversity analysis of rhizosphere bacteria in NT and CM varieties. Table S5: The relative abundances of rhizosphere fungi at the class level between NT and CM varieties. Data were analyzed using the Kruskal–Wallis comparison. “*p* < 0.05” indicates a significant difference between NT and CM varieties. Table S6: The relative abundances of rhizosphere fungi at the order level between NT and CM varieties. Data were analyzed using the Kruskal–Wallis comparison. “*p* < 0.05” indicates a significant difference between NT and CM varieties. Table S7: The relative abundances of rhizosphere fungi between NT and CM varieties at the family level. Data were analyzed using a Kruskal–Wallis comparison. “*p* < 0.05” indicates a significant difference between NT and CM varieties. Table S8: The relative abundances of rhizosphere fungi at the genus level between NT and CM varieties. Data were analyzed using a Kruskal–Wallis comparison. “*p* < 0.05” indicates a significant difference between NT and CM varieties.
