# Supplementary figures and images for: Evaluation of the Ecological Environment Affected by Cry1Ah1 in Poplar
Source: Life (Basel). 2022 Nov 9;12(11):1830. doi: 10.3390/life12111830 (PMC9692618; doi:10.3390/life12111830)

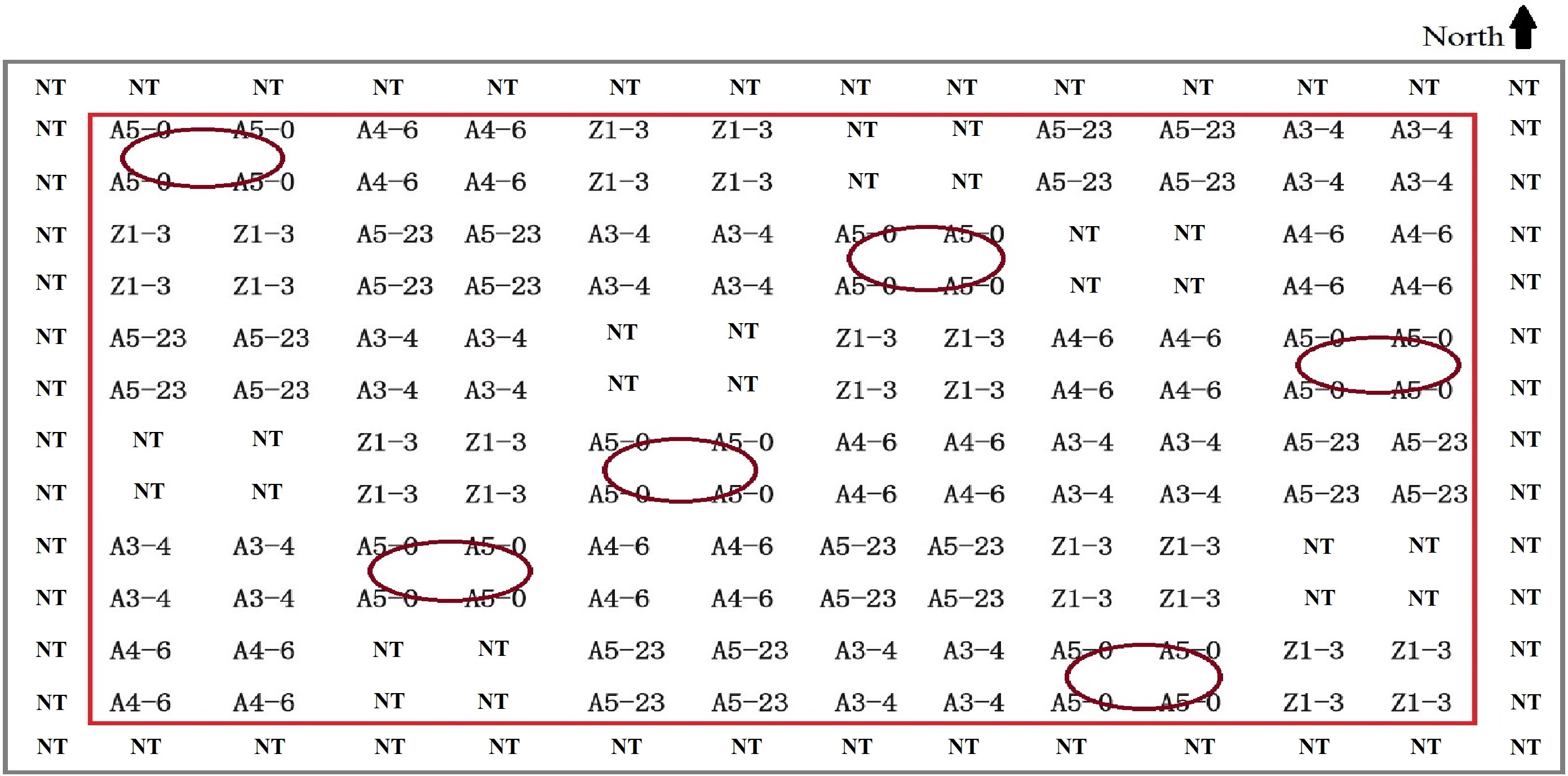

Supplement: Supplementary file 1 [file life-12-01830-s001.zip › supplemental Figure S1.jpg]

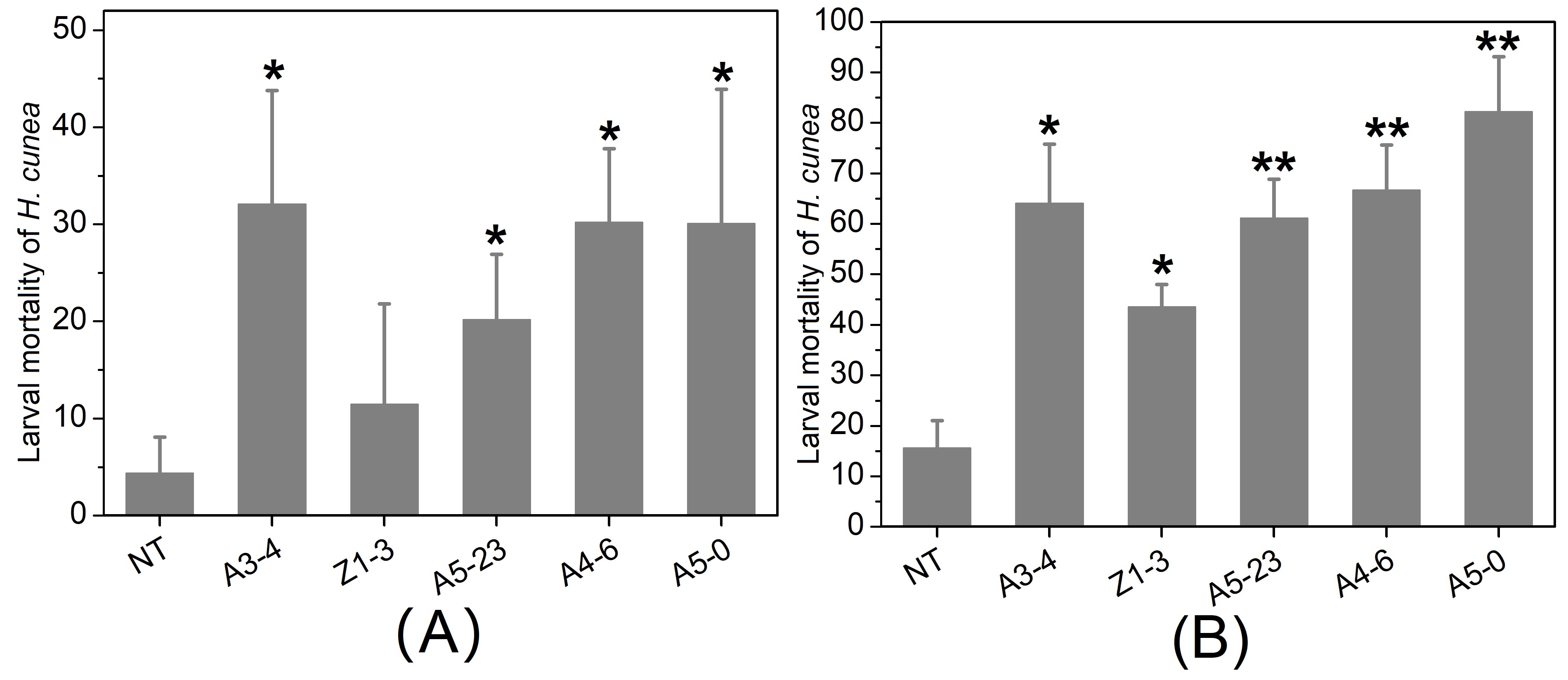

Supplement: Supplementary file 1 [file life-12-01830-s001.zip › supplemental Figure S2.jpg]

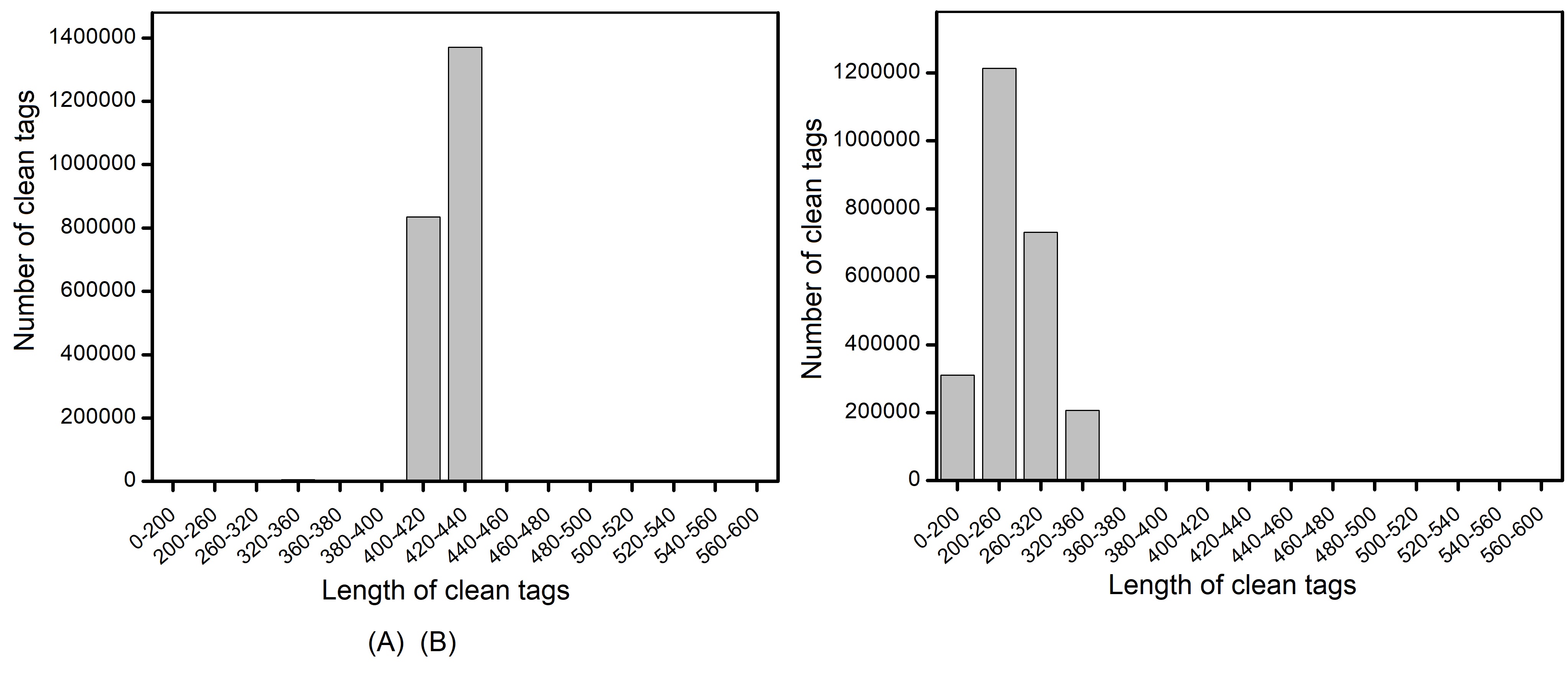

Supplement: Supplementary file 1 [file life-12-01830-s001.zip › supplemental Figure S3.jpg]

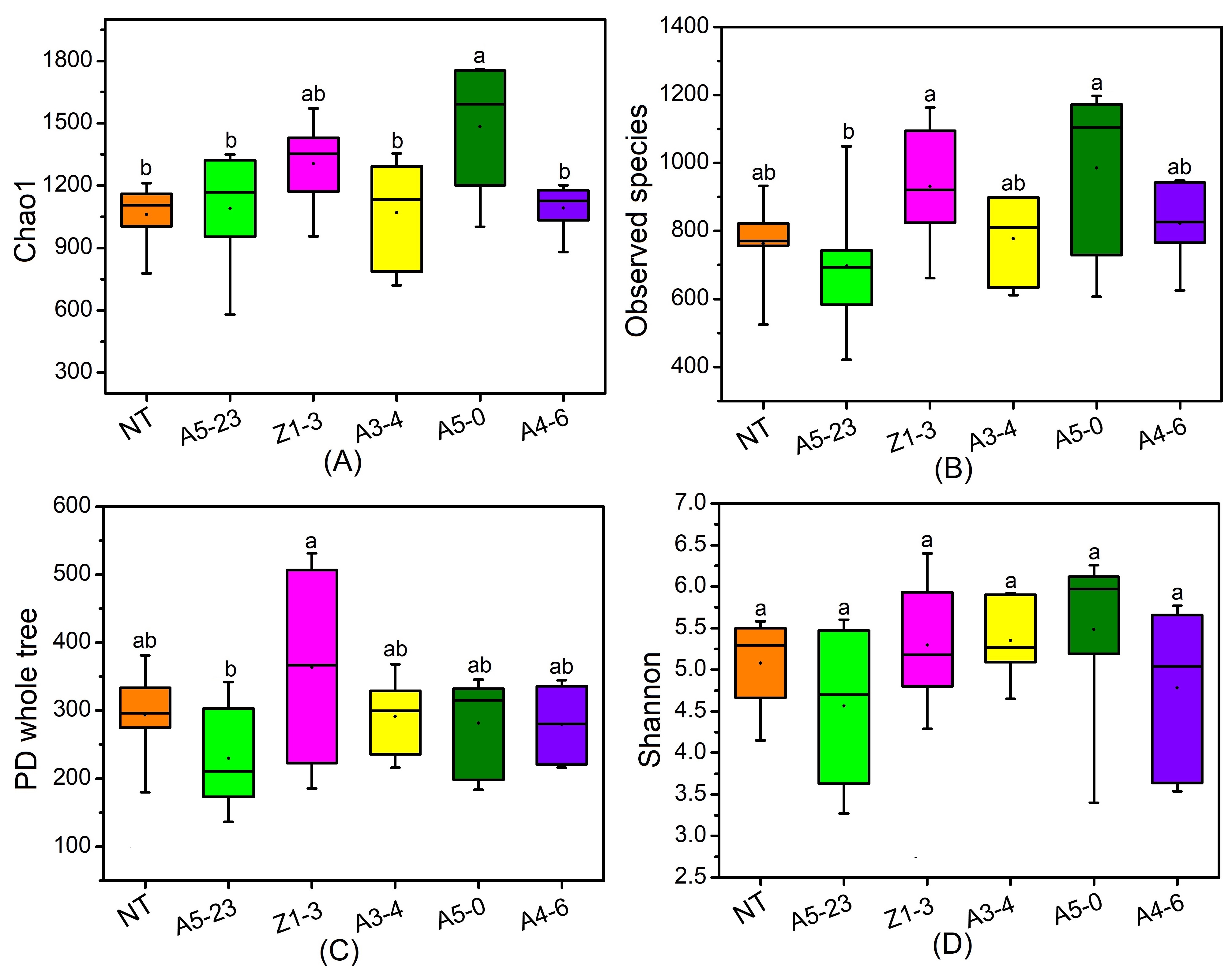

Supplement: Supplementary file 1 [file life-12-01830-s001.zip › supplemental Figure S4.jpg]

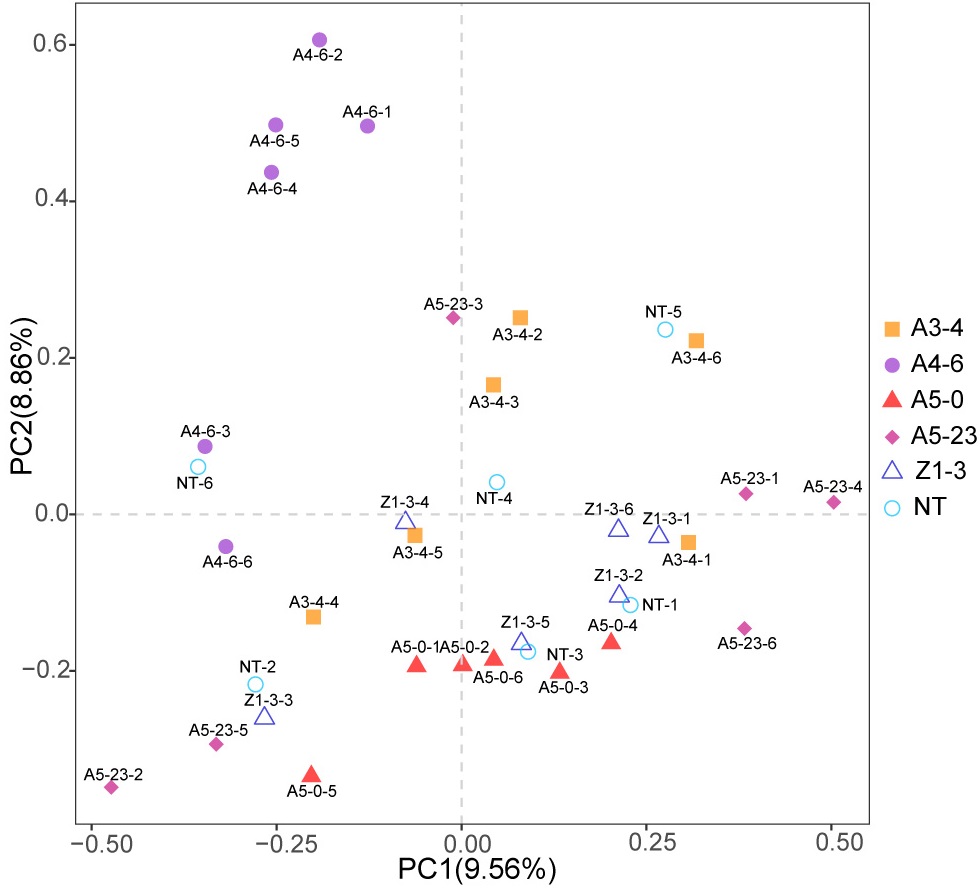

Supplement: Supplementary file 1 [file life-12-01830-s001.zip › supplemental Figure S5.jpg]

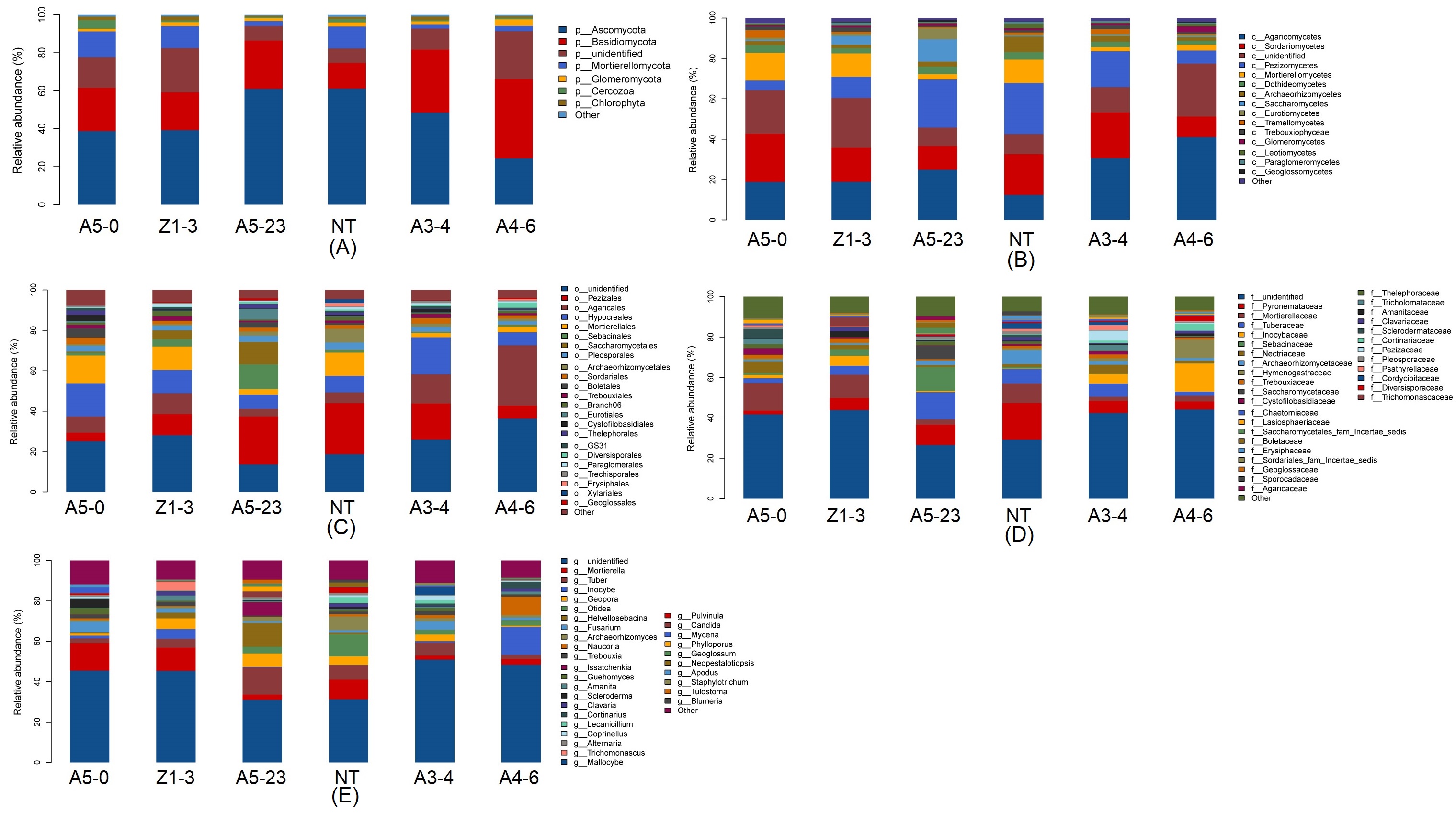

Supplement: Supplementary file 1 [file life-12-01830-s001.zip › supplemental Figure S6.jpg]
